# First-Trimester Assessment of the Extended Fetal Cardiovascular System Using the CASSEAL 3 × 3 Framework: Feasibility, Reproducibility and Diagnostic Performance

**DOI:** 10.3390/jcdd13090422

**Published:** 2026-09-01

**Authors:** Cristina Martínez Payo, Irene García Nieto, Ana Jimena Salcedo Martínez, Carla Salas Gil, Teresa Álvarez Martín, Zita Gambacorti, Pilar Pintado Recarte, Eva Manuela Pena-Burgos, Miguel A. Ortega, Juan De León-Luis, Coral Bravo Arribas

**Affiliations:** 1 Department of Public and Maternal and Child Health, Faculty of Medicine and Health Science, School of Medicine, Complutense University of Madrid, 28040 Madrid, Spain; cmpayo@salud.madrid.org (C.M.P.); irene.nietogci@gmail.com (I.G.N.); jimenasalcedomartinez@gmail.com (A.J.S.M.); carla.salas@salud.madrid.org (C.S.G.); teresa.alvarez@salud.madrid.org (T.Á.M.); ppintadorec@yahoo.es (P.P.R.); corbravo@ucm.es (C.B.A.); 2 Department of Obstetrics and Gynaecology, University Hospital Gregorio Marañón, 28009 Madrid, Spain; 3 Research Pathophysiology of Women, Pregnancy, Childbirth and Puerperium, Instituto de Investigación Sanitaria Gregorio Marañón, 28009 Madrid, Spain; 4 Department of Pathology, University Hospital Gregorio Maranon, 28009 Madrid, Spain; 5 Department of Medicine and Medical Specialities, Faculty of Medicine and Health Sciences, University of Alcalá, 28801 Alcalá de Henares, Spain; miguelangel.ortega@uah.es; 6 Ramón y Cajal Institute of Sanitary Research (IRYCIS), 28034 Madrid, Spain

**Keywords:** fetal echocardiography, first-trimester screening, extended fetal cardiovascular system, prenatal ultrasound, congenital heart disease, CASSEAL, fetal cardiovascular assessment

## Abstract

Background: The Extended Fetal Cardiovascular System (EFCS) encompasses the cardiac, infracardiac, and supracardiac vascular territories that contribute to fetal cardiovascular development and function. The CASSEAL 3 × 3 framework was developed to provide a structured anatomical assessment of the EFCS. This study aimed to evaluate its feasibility, reproducibility, and diagnostic performance during first-trimester screening. Methods: In this prospective single-center study, singleton pregnancies undergoing ultrasound examination between 11 + 0 and 13 + 6 weeks of gestation were assessed using the CASSEAL 3 × 3 framework. Feasibility, examination time, use of complementary techniques, interobserver reproducibility, and diagnostic performance were evaluated. Prenatal follow-up and postnatal findings served as the reference standard. Results: A total of 288 pregnancies were included in the final analysis. Complete visualization of all nine axial views was achieved in 86.5% of examinations. Mean examination time was 12.5 ± 3.3 min. Higher gestational age increased the likelihood of complete visualization, whereas higher maternal body mass index reduced feasibility. Interobserver agreement was high, with no significant systematic differences between operators. Six suspected EFCS abnormalities were identified during first-trimester examination. Diagnostic performance demonstrated high specificity (99.64%) and negative predictive value (98.58%), whereas sensitivity was moderate and should be interpreted cautiously due to the limited number of confirmed abnormalities. Conclusions: The CASSEAL 3 × 3 framework is a feasible and reproducible approach for structured first-trimester assessment of the EFCS. Diagnostic performance estimates should be interpreted cautiously given the limited number and spectrum of confirmed abnormalities and the use of a composite reference standard without systematic postnatal echocardiography. The framework may be better suited to specialized fetal medicine settings or as an adjunct to routine first-trimester assessment rather than as a universal screening tool.

## 1. Introduction

Congenital heart defects (CHD) are among the most common congenital anomalies, with an estimated prevalence ranging from 4 to 13 per 1000 live births and remain a major cause of neonatal morbidity and mortality [1,2]. Despite substantial advances in ultrasound technology, standardized screening protocols, and specialized training programs, prenatal detection rates continue to show considerable variability across healthcare settings and remain suboptimal in many populations [2,3,4]. Early identification of cardiovascular abnormalities is essential to optimize prenatal counselling, pregnancy surveillance, delivery planning, and postnatal management [1]. Consequently, international scientific societies such as the International Society of Ultrasound in Obstetrics and Gynecology (ISUOG) have progressively expanded recommendations for fetal cardiac screening beyond the traditional four-chamber view, promoting a more systematic evaluation of the fetal cardiovascular anatomy through multiple axial planes and outflow tract assessment [5,6].

Although current screening protocols have substantially improved prenatal detection of congenital heart disease, most remain primarily focused on intracardiac anatomy and the evaluation of the ventricular outflow tracts [1,7]. Over recent decades, several structured approaches based on sequential axial views have been incorporated into routine fetal cardiac assessment, including the extended cardiac echography examination (ECEE), which has demonstrated high diagnostic performance for the prenatal detection of congenital heart disease [8]. However, these protocols are largely designed to assess cardiac morphology and great vessel anatomy and do not systematically integrate other vascular, mediastinal, and hepato-splanchnic structures that may provide relevant information regarding fetal cardiovascular development, pathology, and the broader fetal cardiovascular system.

Within this context, the concept of the Extended Fetal Cardiovascular System (EFCS) emerged as a broader anatomical and functional framework encompassing not only the fetal heart and great arteries, but also the adjacent vascular, mediastinal and hepato-splanchnic territories that participate in cardiovascular development and fetal circulation. Several structures within these territories, including the fetal thymus (“thy-box”) [9,10], aberrant right subclavian artery [11], aortic arch anatomy [12], persistent left superior vena cava [13], and portal venous circulation represented by the persistent right umbilical vein [14], have demonstrated diagnostic relevance in the prenatal evaluation of cardiovascular abnormalities and associated conditions. This integrated perspective supports the concept that fetal cardiovascular assessment should extend beyond isolated cardiac examination toward a more comprehensive evaluation of interconnected anatomo-functional territories.

**Figure 1:**
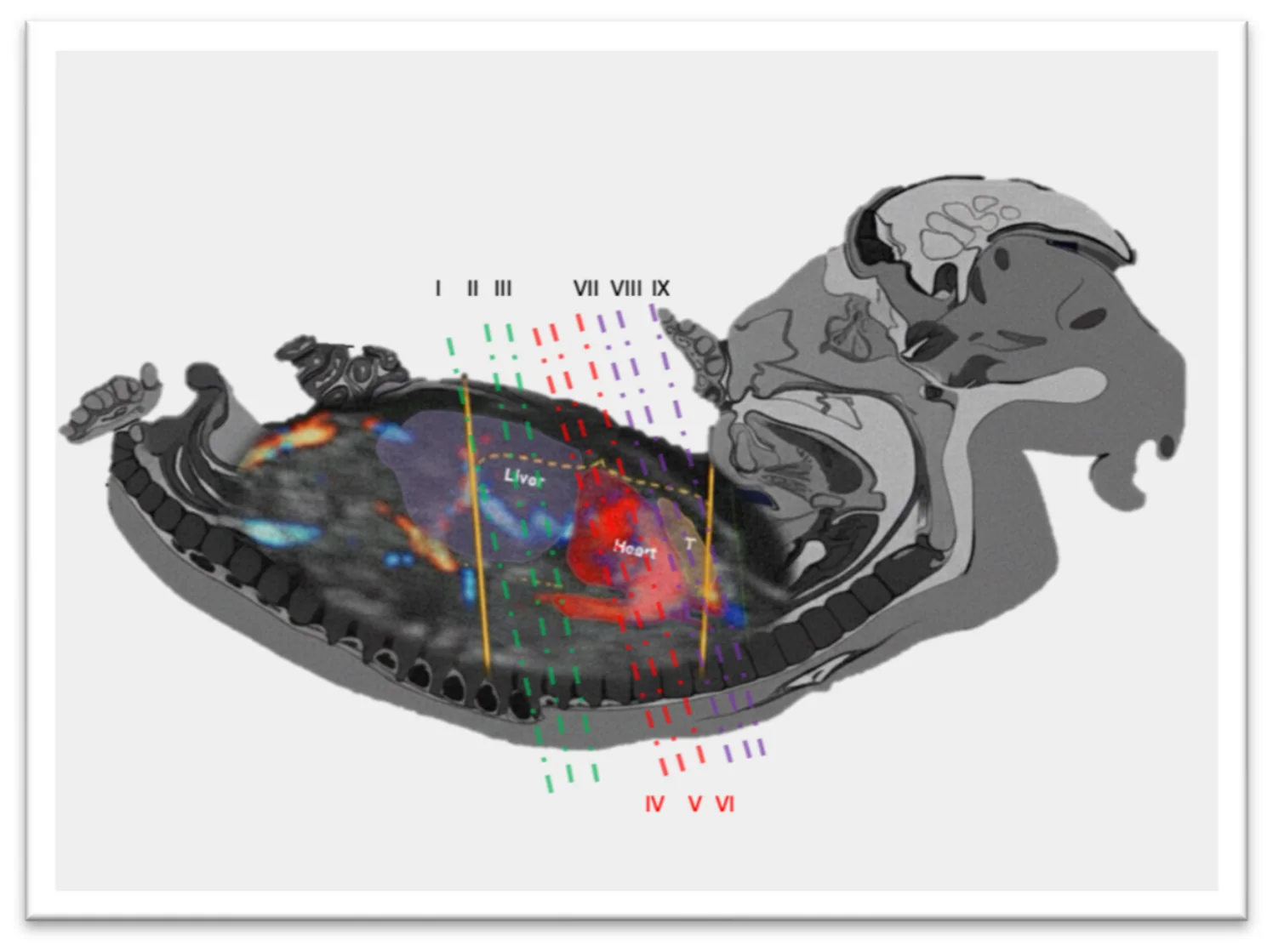
Sagittal representation of the Extended Fetal Cardiovascular System (EFCS) and distribution of the nine standardized axial ultrasound views of the Cardiovascular System Sonographic Evaluation Algorithm (CASSEAL) in a 12-week fetus (CRL ≈ 60 mm). Green indicates the infracardiac territory, red the cardiac territory, and purple the supracardiac territory.

Within this conceptual framework, the Cardiovascular System Sonographic Evaluation Algorithm (CASSEAL) was developed as a structured ultrasound approach for the systematic assessment of the Extended Fetal Cardiovascular System (EFCS), conceived as a continuous anatomo-functional unit [5]. Figure 1 illustrates the EFCS concept in a fetus of approximately 12 weeks’ gestation (CRL ≈ 60 mm), in which the infracardiac, cardiac and supracardiac territories are contained within an anatomical distance of approximately 12 mm, integrating the hepato-splanchnic circulation, the heart, and the adjacent mediastinal vascular structures along a single anatomical axis. To facilitate a systematic evaluation of this continuum, the CASSEAL algorithm organizes the EFCS into three integrated anatomo-functional territories—infracardiac, cardiac and supracardiac—each explored through three consecutive axial planes, resulting in a standardized 3 × 3 framework composed of nine sequential views. This organization allows the assessment of intracardiac anatomy together with adjacent vascular, mediastinal and hepato-splanchnic structures within a single structured examination. Previous studies demonstrated high feasibility and reproducibility of this approach in the second trimester [5], while subsequent magnetic resonance imaging studies further supported the EFCS as an integrated anatomical model for prenatal cardiovascular assessment [6].

More recently, the CASSEAL framework was adapted to first-trimester ultrasound, demonstrating the anatomical feasibility of reproducing the same structured sequential assessment of the EFCS during early pregnancy [15]. However, although feasibility represents an essential first step, it does not necessarily imply clinical applicability. The feasibility, reproducibility, and diagnostic performance of this structured 3 × 3 framework during first-trimester screening have not been adequately evaluated. Furthermore, the ability of the CASSEAL approach to identify abnormalities involving different territories of the EFCS during early gestation remains largely unknown. Therefore, the present study was designed to clinically validate the CASSEAL 3 × 3 framework for first-trimester assessment of the Extended Fetal Cardiovascular System by evaluating its feasibility, reproducibility, and diagnostic performance in an unselected population.

## 2. Materials and Methods

### 2.1. Study Design

This was a prospective, single-center observational study designed to evaluate the feasibility, reproducibility, and diagnostic performance of the CASSEAL 3 × 3 framework for first-trimester assessment of the Extended Fetal Cardiovascular System (EFCS).

Participants were pregnant women attending the first-trimester clinic between October 2022 and December 2024. The study was approved by the local Ethics Committee (study code ALEESCA1T), and participation required that all patients be of legal age and provide written informed consent.

The study design included two distinct analytical components:

Main validation cohort: Evaluation of a consecutive cohort examined by an expert operator to determine baseline visualization rates and diagnostic performance of the algorithm.

Interobserver agreement subanalysis: Blinded double evaluation in a subsample to compare feasibility and technical dependence between different levels of experience.

The expert operator was a fetal medicine specialist with more than 25 years of experience in fetal echocardiography and extensive familiarity with the CASSEAL algorithm and was responsible for evaluation of the full cohort for performance analysis. The junior operator was a specialist with 5 years of experience in obstetric ultrasound, including first-trimester fetal cardiovascular imaging, but without previous experience with the CASSEAL 3 × 3 framework. Both operators received specific training in the CASSEAL methodology and followed the standardized acquisition protocol previously described [5]. Prior to study initiation, both operators underwent a structured calibration process consisting of six supervised CASSEAL examinations evaluated by an independent supervisor to ensure adherence to the standardized acquisition protocol before participation in the study.

For the comparative subanalysis, a sample of 50 pregnant women was selected, who were examined consecutively and independently by both operators (blinded to each other’s findings), with the aim of comparing visualization rates by view, the need for additional technical resources, and diagnostic agreement.

### 2.2. Study Population

Singleton pregnancies with a crown–rump length corresponding to a gestational age between 11 + 0 and 13 + 6 weeks were eligible for inclusion. The study population was intended to represent a routine first-trimester screening cohort within the predefined eligibility criteria. Recruitment was determined by the investigator’s availability within the routine first-trimester clinic schedule. Patients attended the clinic on different days and during morning or afternoon shifts according to standard appointment allocation, whereas the investigator did not cover all clinical shifts. Consequently, participation could only be offered to eligible women attending during investigator-covered sessions. No maternal, fetal, or ultrasound characteristics were used to determine recruitment according to investigator availability.

Exclusion criteria included multiple pregnancies and conditions that precluded reliable assessment of the Extended Fetal Cardiovascular System (EFCS), such as severe multisystem malformations, marked anatomical distortion, fetal hydrops, or nuchal translucency above the 95th percentile. Fetuses with markedly increased nuchal translucency were excluded because of their high association with chromosomal abnormalities, major multisystem malformations, hydrops fetalis, spontaneous fetal loss, and pregnancy termination, factors that could compromise both image acquisition and the establishment of a complete prenatal and postnatal reference standard.

Baseline maternal variables (age, weight, height, body mass index, presence of uterine pathology or anomalies), gestational variables (type of pregnancy and gestational age determined by crown–rump length), ultrasound data (CRL in mm, nuchal translucency percentile for CRL), and perinatal data (mode of delivery, gestational age at delivery, neonatal sex, birth weight, Apgar score at 1 and 5 min, umbilical cord arterial pH, and need for neonatal admission) were recorded.

Newborns were followed for up to 1 year of life to assess the occurrence of pathology related to the study outcomes. Data were obtained from clinical interviews and electronic medical records; in selected cases, patients were contacted by telephone to complete missing information.

### 2.3. Ultrasound Procedure (First-Trimester CASSEAL Algorithm) [15]

**Figure 2:**
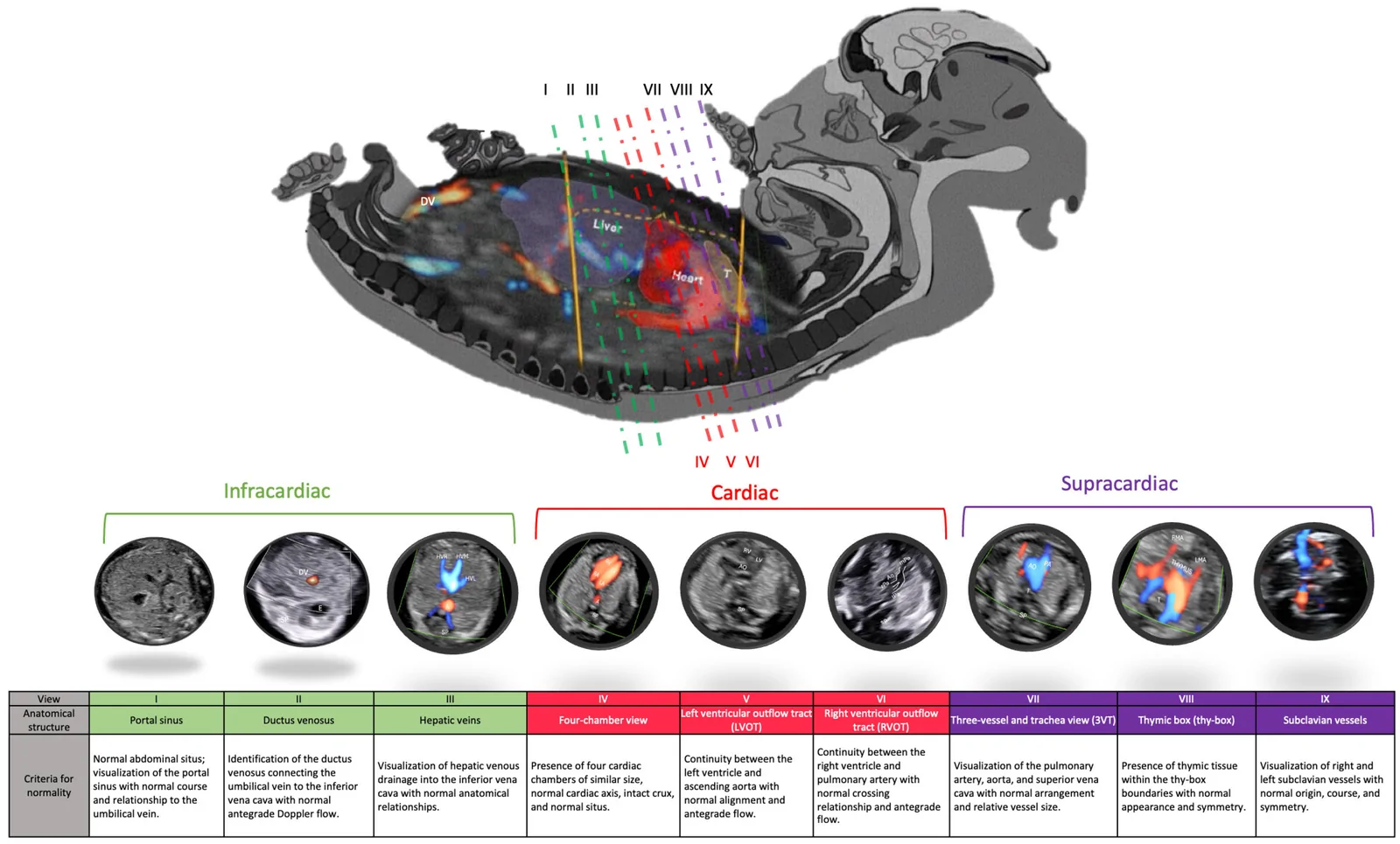
The CASSEAL 3 × 3 framework for first-trimester assessment of the Extended Fetal Cardiovascular System (EFCS): anatomical organization, standardized axial views, and normality criteria. Green indicates the infracardiac territory, red the cardiac territory, and purple the supracardiac territory.

All patients underwent evaluation using the CASSEAL algorithm adapted to the first trimester (Figure 2). The CASSEAL framework evaluates the Extended Fetal Cardiovascular System (EFCS) through three anatomo-functional territories (infracardiac, cardiac, and supracardiac), each explored by three sequential axial views, resulting in a standardized 3 × 3 assessment protocol. This systematic caudo–cranial examination comprises nine axial planes organized according to these three territories:

Infracardiac zone: Portal sinus (I), ductus venosus (II), and hepatic veins (III), which also allow assessment of situs, aorta, and inferior vena cava.

Cardiac zone: Four-chamber view (IV), left ventricular outflow tract (V), and right ventricular outflow tract (VI).

Supracardiac zone: Three-vessel and trachea view (VII), thymic box (“thy-box”) (VIII), and subclavian vessels (IX).

Initial assessment was performed using the transabdominal approach with a 4–8 MHz convex probe (Samsung HERA W10^®^, Samsung Medison Co., Ltd., Seoul, Republic of Korea), whenever adequate visualization was achievable. A stepwise image optimization protocol was applied, including B-mode optimization, use of color Doppler, and complementary transvaginal examination when required to obtain adequate visualization.

### 2.4. Feasibility and Technical Variables

For each of the nine views and for the overall examination, the following variables were recorded:

Visualization (Yes/No): Ability to obtain a satisfactory diagnostic image.

Need for color Doppler: Mandatory use of color Doppler to identify or validate the anatomical structure.

Need for transvaginal probe: Requirement of transvaginal approach to obtain the view.

Overall feasibility: Ability to obtain a satisfactory diagnostic image in all nine views (Yes/No).

### 2.5. Definition of Normality

Anatomical normality for each view was defined according to previously published first-trimester criteria for the corresponding anatomical structure [15]. Assessment included the presence, visualization quality, and correct anatomical relationships of the expected structures, vessel course and symmetry, cardiac chamber configuration, alignment of the outflow tracts, and normal antegrade flow patterns when Doppler examination was required. Detailed normality criteria for each of the nine CASSEAL views are provided in Supplementary Table S1.

### 2.6. Follow-Up and Reference Standard

Given that some abnormalities of the Extended Fetal Cardiovascular System (EFCS) may not be fully characterized during the first trimester, all suspected findings were evaluated using a composite reference standard based on prenatal and postnatal follow-up.

All ongoing pregnancies underwent detailed second-trimester anatomical ultrasound examination. Additional prenatal follow-up, including third-trimester ultrasound assessment when available, was reviewed to identify abnormalities involving the EFCS. After birth, all newborns underwent routine clinical examination by a neonatologist, and neonatal medical records were reviewed for study outcomes. In cases with prenatal suspicion of an EFCS abnormality, abnormal findings on neonatal examination, or other clinical indications, targeted postnatal assessment, including echocardiography performed by an experienced pediatric echocardiographer, was undertaken. In cases of pregnancy termination or fetal loss, available prenatal findings were correlated with autopsy or pathological examination when available.

Diagnostic performance of the CASSEAL algorithm was assessed by comparing first-trimester findings with the final diagnosis established through this composite reference standard.

### 2.7. Statistical Analysis

Statistical analysis was performed using the R environment 4.6.0 (R Foundation for Statistical Computing, Vienna, Austria). Categorical variables were expressed as absolute frequencies and percentages, whereas continuous variables were summarized as mean ± standard deviation.

Feasibility of the CASSEAL 3 × 3 framework was assessed by calculating visualization rates for each individual axial view and for complete visualization of all nine views. Exact 95% confidence intervals were estimated for proportions.

To identify factors associated with complete visualization of the CASSEAL 3 × 3 framework, univariable and multivariable binary logistic regression analyses were performed using complete visualization of all nine views as the dependent variable. Maternal age, gestational age at ultrasound, body mass index, and uterine anomalies were evaluated as explanatory variables. Maternal age, gestational age at ultrasound, and body mass index were entered as continuous variables, whereas uterine anomalies were entered as a binary variable. All four explanatory variables were included simultaneously in the multivariable model. Results were expressed as odds ratios (OR) with 95% confidence intervals. There were no missing data for the variables included in the primary feasibility, reproducibility, regression, or diagnostic performance analyses; therefore, no imputation procedures were required. No formal a priori sample size calculation was performed. The sample size was determined by the number of eligible participants recruited during the predefined prospective study period.

Diagnostic performance of the first-trimester CASSEAL algorithm was evaluated using the composite prenatal and postnatal reference standard described above. Sensitivity, specificity, positive predictive value (PPV), and negative predictive value (NPV), with 95% confidence intervals, were calculated for the detection of abnormalities involving the Extended Fetal Cardiovascular System. Diagnostic performance was assessed at the patient level, with the presence of any EFCS abnormality as the diagnostic outcome. Accordingly, a first-trimester CASSEAL examination classified as abnormal was considered a true positive when any EFCS abnormality was subsequently confirmed by the reference standard, irrespective of concordance between the initially suspected anatomical finding or EFCS territory and the final diagnosis.

A two-sided *p* value < 0.05 was considered statistically significant.

## 3. Results

**Figure 3:**
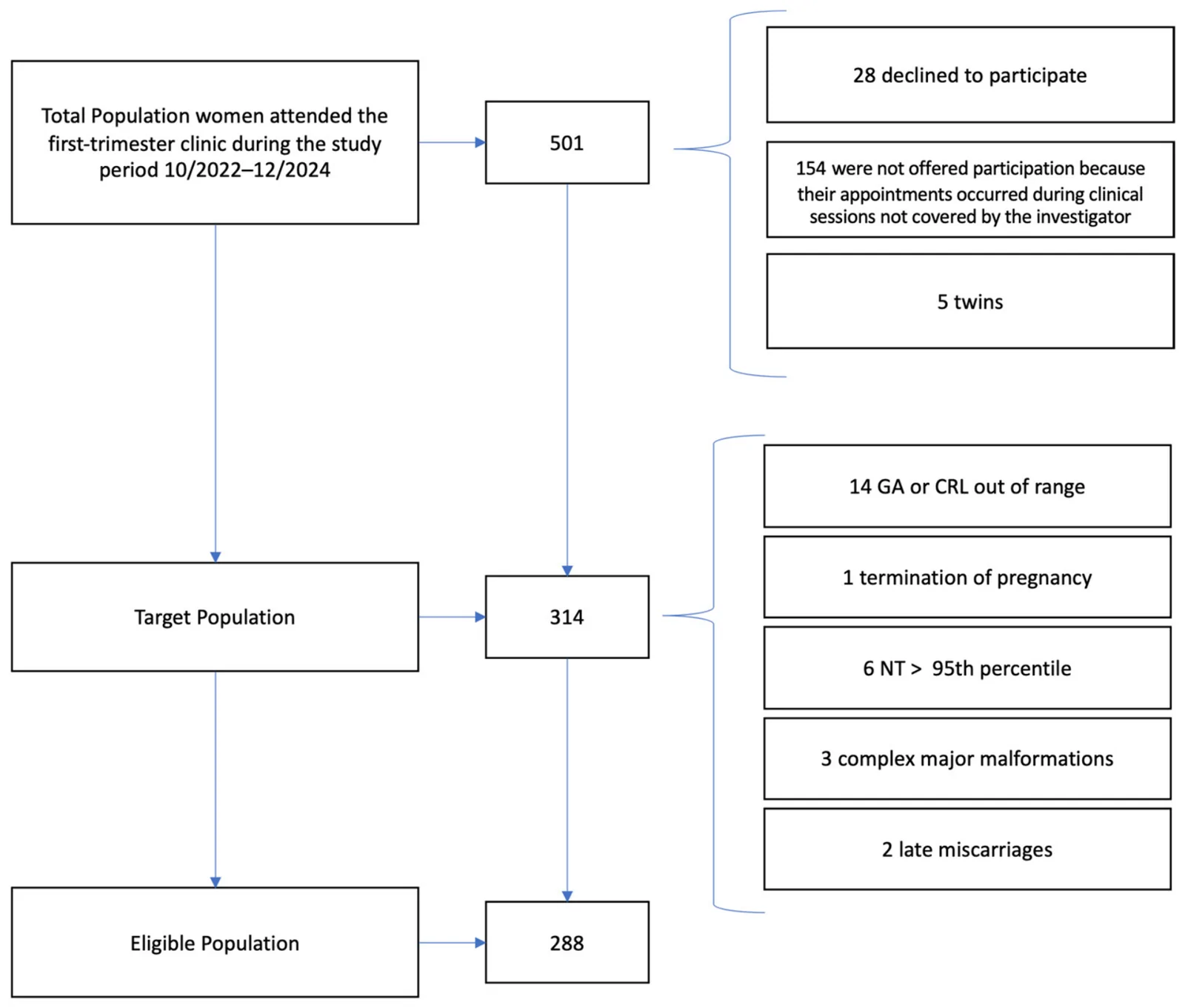
Flow diagram of study population selection.

During the study period, 501 women attended the first-trimester clinic. Of these, 154 could not be offered participation because their scheduled appointments occurred during clinical sessions not covered by the investigator, 28 declined participation and five had multiple pregnancies. The resulting target population comprised 314 singleton pregnancies. Of these, 26 were excluded according to the predefined criteria: 14 because crown–rump length corresponded to a gestational age outside the predefined eligibility range, six because nuchal translucency was above the 95th percentile, three because of major complex fetal malformations, one because of pregnancy termination, and two because of late pregnancy loss. The final study population therefore comprised 288 singleton pregnancies (Figure 3).

Baseline maternal, gestational, ultrasound, and perinatal characteristics are summarized in Table 1. The mean maternal age was 33.7 ± 5.1 years and the mean body mass index was 25.0 ± 4.8 kg/m^2^. Ultrasound examinations were performed at a mean gestational age of 13.1 ± 0.7 weeks, corresponding to a mean crown-rump length of 67.8 ± 8.5 mm. Perinatal outcomes were globally favorable, with a mean gestational age at delivery of 38.7 ± 4.9 weeks and a cesarean section rate of 25.3%.

**Figure 4:**
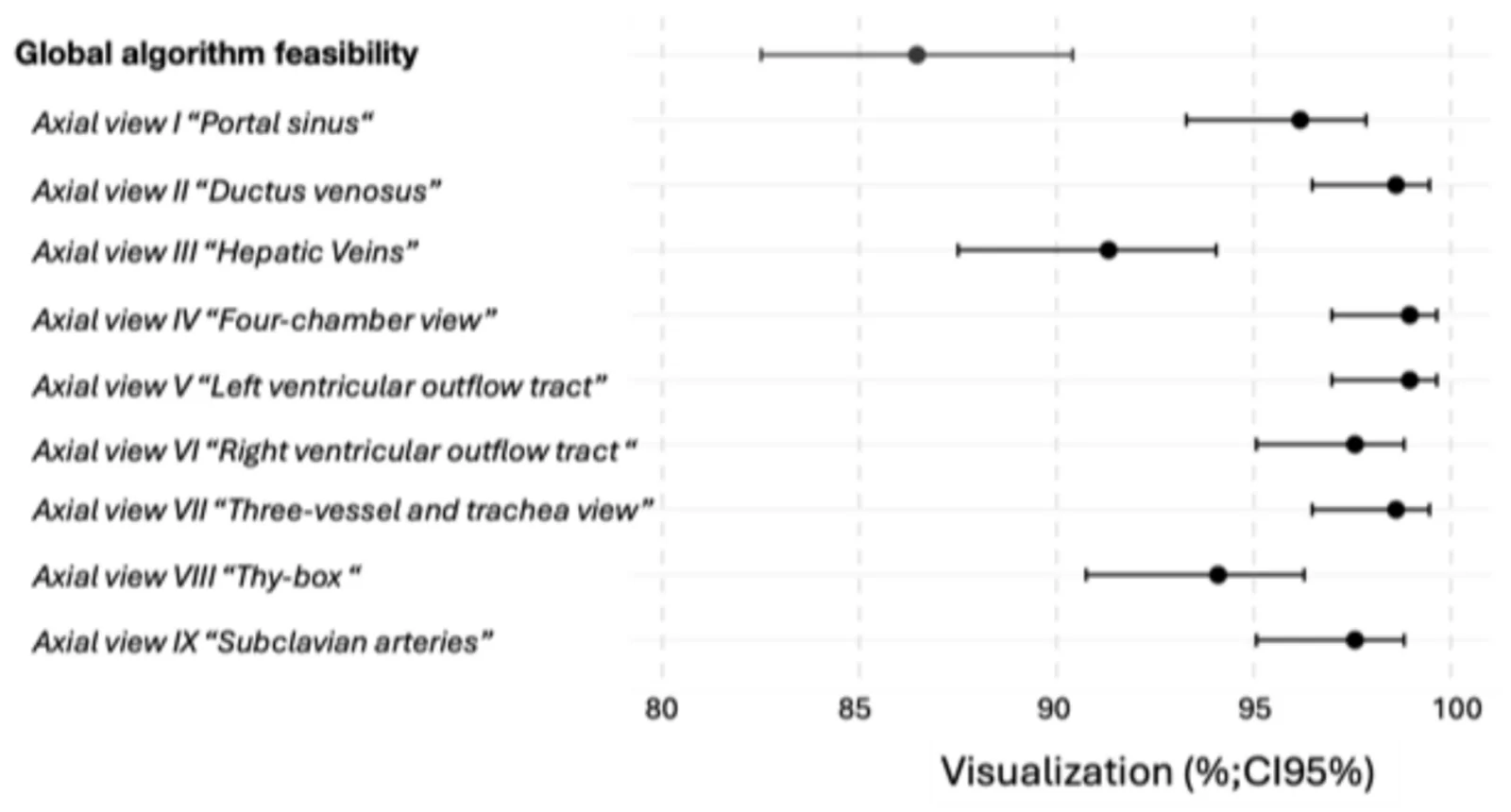
Proportion of visualization for each CASSEAL axial view in the first trimester and Global algorithm feasibility, with 95% confidence intervals.

Feasibility of the CASSEAL 3 × 3 framework was high. Complete visualization of all nine standardized views was achieved in 86.5% of examinations. Visualization rates for individual views ranged from 91.3% to 99.0%, with values above 94% in seven of the nine planes. Figure 4 summarizes visualization rates and corresponding 95% confidence intervals for each view and for complete visualization of the entire framework. Although visualization rates for individual views were consistently high, the requirement to obtain all nine planes reduced the overall proportion of complete examinations, reflecting the technical demands of the full CASSEAL protocol.

Use of complementary techniques varied substantially across the different CASSEAL views (Table 1). Color Doppler was required in 100% of examinations for assessment of the thy-box and in more than 85% of examinations for evaluation of the ductus venosus and hepatic veins, whereas its use was considerably lower for conventional cardiac views. Similarly, the transvaginal approach was used selectively according to the anatomical plane being assessed, with utilization rates ranging from 34.7% to 45.8%. Overall, combined transabdominal and transvaginal examination was required in 54.6% of cases. The mean examination time was 12.5 ± 3.3 min.

Factors associated with complete visualization of the CASSEAL 3 × 3 framework are summarized in Table 2. In univariable analysis, higher gestational age and lower maternal body mass index were significantly associated with successful visualization of all nine views. After multivariable adjustment, both variables remained independently associated with complete visualization. Specifically, increasing gestational age was associated with higher odds of complete assessment of the EFCS, whereas increasing maternal body mass index was associated with reduced feasibility. Maternal age and the presence of uterine anomalies were not significantly associated with complete visualization.

Interobserver agreement analysis is summarized in Table 3. No statistically significant differences were observed between senior and junior operators in complete visualization of the CASSEAL 3 × 3 framework or in any individual axial view. Visualization rates were consistently high in both operators, exceeding 84% for all views and reaching 100% for several cardiac planes.

Overall agreement was high across the entire framework, with a percentage agreement of 76% for complete visualization and values ranging from 80% to 100% for individual views. Cohen’s kappa coefficients showed variable results, ranging from moderate agreement for complete visualization and selected views to low or null values in planes characterized by extremely high visualization rates. Negative or near-zero kappa values should be interpreted cautiously because of the extremely low prevalence of the “non-visualized” category. This finding reflects the well-recognized limitations of kappa statistics in situations with highly unbalanced category distributions and should be interpreted in the context of the consistently high observed agreement between operators. McNemar’s test showed no statistically significant differences between operators for complete visualization of the CASSEAL framework or for any individual view (all *p* > 0.05). These findings indicate that discordant classifications were not systematically attributable to either operator and support the reproducibility of the framework despite the low kappa values observed in selected views.

Abnormal findings involving the Extended Fetal Cardiovascular System (EFCS) were suspected during first-trimester assessment in six cases (2.1%). These findings involved different anatomo-functional territories of the CASSEAL framework and included four cases of aberrant right subclavian artery (ARSA) within the supracardiac territory, one suspected persistent right umbilical vein (PRUV) within the infracardiac territory, and one suspected abnormality involving the cardiac outflow tract.

Follow-up evaluation identified a total of nine confirmed EFCS abnormalities (3.1%). All four ARSA cases detected during the first trimester were confirmed during subsequent prenatal and postnatal assessment. In one additional fetus, the first-trimester CASSEAL examination was classified as abnormal because of a suspected cardiac outflow-tract anomaly. Although this specific finding was not confirmed during follow-up, a persistent right umbilical vein (PRUV) was subsequently identified in the same fetus. Accordingly, this case was classified as a true positive in the patient-level analysis of any EFCS abnormality, although the anatomical finding and EFCS territory identified at first-trimester assessment were discordant with the final diagnosis. Four ventricular septal defects were subsequently identified in fetuses classified as normal at the first-trimester CASSEAL examination and therefore represented false-negative examinations. The PRUV suspected during first-trimester assessment in another fetus was not confirmed during follow-up and represented the single false-positive examination. Classification of all suspected and confirmed EFCS abnormalities is summarized in Table 4.

Diagnostic performance of the first-trimester CASSEAL algorithm is summarized in Table 5. Overall specificity and negative predictive value were high (99.6% and 98.6%, respectively). However, these estimates should be interpreted cautiously given the small number of confirmed EFCS abnormalities and the characteristics of the composite reference standard. Sensitivity was moderate, with four false-negative examinations corresponding to ventricular septal defects.

Finally, Table 6 compares the original single-center CASSEAL study published in 2015 with the present first-trimester clinical validation study. Despite being performed approximately seven weeks earlier in gestation (13.1 vs. 20.0 weeks), the CASSEAL 3 × 3 framework maintained high feasibility, achieving complete visualization in 86.5% of examinations, exceeding the overall feasibility reported in the original study. Visualization rates remained above 90% for all individual views and improved for selected planes, particularly the thy-box (View VIII). Earlier application of the framework required longer examination times and more frequent use of complementary techniques, including color Doppler and transvaginal ultrasound. In contrast to the original study, which focused primarily on feasibility and reproducibility, the present study additionally evaluated diagnostic performance and clinical applicability, providing the first clinical validation of the CASSEAL framework during first-trimester assessment of the Extended Fetal Cardiovascular System.

### Figures, Tables and Schemes

The figure illustrates the EFCS concept as a continuous anatomo-functional unit extending between the two yellow lines over an anatomical distance of approximately 12–14 mm. Within this segment, the hepato-splanchnic, cardiac, and mediastinal vascular territories are integrated along a single anatomical axis, forming the basis of the CASSEAL 3 × 3 framework.

The nine standardized axial views of the CASSEAL algorithm are distributed from caudal to cranial and grouped into three anatomo-functional territories:

Infracardiac territory (green):

- View I: Portal sinus
- View II: Ductus venosus
- View III: Hepatic veins

Cardiac territory (red):

- View IV: Four-chamber view
- View V: Left ventricular outflow tract (LVOT)
- View VI: Right ventricular outflow tract (RVOT)

Supracardiac territory (purple):

- View VII: Three-vessel and trachea view (3VT)
- View VIII: Thymic box (thy-box)
- View IX: Subclavian vessels

Representative first-trimester ultrasound images corresponding to the nine sequential axial views of the CASSEAL algorithm. The views are organized into three anatomo-functional territories: the infracardiac territory (Views I–III: portal sinus, ductus venosus, and hepatic veins), the cardiac territory (Views IV–VI: four-chamber view, left ventricular outflow tract, and right ventricular outflow tract), and the supracardiac territory (Views VII–IX: three-vessel and trachea view, thymic box, and subclavian vessels). Together, these views constitute the standardized CASSEAL 3 × 3 framework for systematic first-trimester assessment of the Extended Fetal Cardiovascular System.

The central illustration depicts the anatomical organization of the EFCS and the spatial distribution of the nine sequential axial planes. Representative ultrasound examples of each examination view are displayed below and grouped according to their corresponding anatomo-functional territory. The lower panel summarizes the anatomical structure assessed in each view and the criteria used to define normal findings during first-trimester examination.

## 4. Discussion

The present study provides the first clinical validation of the CASSEAL 3 × 3 framework during first-trimester assessment of the Extended Fetal Cardiovascular System (EFCS). The results demonstrate high feasibility and good reproducibility, supporting the clinical applicability of a structured anatomo-functional approach to fetal cardiovascular assessment during early pregnancy. Diagnostic performance estimates should be interpreted cautiously given the limited number of confirmed abnormalities and the characteristics of the reference standard.

This broader perspective is rooted in the original CASSEAL concept, first described in 2015 as a structured sequential approach for evaluation of the fetal cardiovascular system through nine standardized axial views [5]. Subsequent investigations expanded this concept by exploring cardiovascular structures beyond the fetal heart itself, including the fetal thymus, aortic arch anatomy, supra-aortic vessels, systemic and umbilical venous circulation, and their integration within the concept of the Extended Fetal Cardiovascular System [6,9,10,11,12,13,14]. The present study extends this line of research by demonstrating that these anatomo-functional territories can be systematically assessed during the first trimester using a unified 3 × 3 framework.

In an unselected cohort of 288 pregnant women, complete visualization of all nine CASSEAL views was achieved in 86.5% of examinations, with individual visualization rates exceeding 90% in all planes (Table 1, Figure 4). These findings confirm that systematic assessment of the Extended Fetal Cardiovascular System (EFCS) is technically feasible during the first trimester, despite the small anatomical dimensions and inherent imaging challenges of early gestation. This observation is particularly relevant because the CASSEAL framework extends beyond conventional fetal cardiac screening, incorporating infracardiac, cardiac, and supracardiac territories into a single structured examination (Figure 1 and Figure 2). The ability to reproducibly assess these three anatomo-functional territories supports the concept that the fetal cardiovascular system can be evaluated as an integrated continuum rather than as an isolated cardiac structure.

From this perspective, the differences observed between individual CASSEAL views should be interpreted in the context of the anatomical organization of the EFCS. Views traditionally incorporated into routine fetal cardiac screening, such as the four-chamber view and the ventricular outflow tracts (Views IV–VI), demonstrated visualization rates approaching 100% (Table 1), reflecting their long-standing integration into obstetric ultrasound practice and their inclusion in current international screening recommendations [1,16]. In contrast, views exploring the infracardiac and supracardiac territories—including hepatic venous circulation, the thymic box, and the supra-aortic vessels—showed lower feasibility and greater dependence on complementary techniques (Table 1, Figure 4). These structures are less frequently incorporated into conventional screening protocols despite their recognized diagnostic value in the assessment of fetal cardiovascular development and pathology [9,10,11,12,13,14].

This pattern likely reflects, at least in part, the broader anatomical scope of the algorithm. Unlike conventional fetal cardiac screening protocols, which focus primarily on intracardiac anatomy and the great vessels, the CASSEAL framework was specifically designed to evaluate the Extended Fetal Cardiovascular System as a continuum encompassing infracardiac, cardiac, and supracardiac territories [5,6]. Consequently, the lower feasibility observed in some planes reflects the inclusion of anatomical structures that are not routinely assessed during standard examinations rather than an intrinsic limitation of the method itself. Importantly, despite this expanded scope, visualization rates remained above 90% in all nine views (Table 1, Figure 4), supporting the feasibility of incorporating these additional territories into structured first-trimester assessment.

Analysis of factors associated with complete visualization of the CASSEAL 3 × 3 framework provides relevant practical information for clinical implementation (Table 2). As observed in the original CASSEAL study [5], feasibility was influenced by technical and anatomical factors that affect ultrasound image acquisition. In the present first-trimester cohort, increasing gestational age was independently associated with higher odds of complete visualization, whereas higher maternal body mass index reduced the probability of successfully completing the entire examination. These findings suggest that, despite the earlier gestational age and smaller anatomical dimensions, the determinants of feasibility remain largely consistent with those previously described for second-trimester application of the algorithm. The positive effect of gestational age is biologically plausible, as progressive fetal growth during the first trimester facilitates identification of the anatomical landmarks required for completion of the CASSEAL protocol. Conversely, increasing maternal body mass index may reduce image quality through greater ultrasound attenuation, a limitation that has been consistently reported across obstetric ultrasound examinations.

Interobserver reproducibility represents one of the most relevant attributes of a structured screening algorithm. Similar to the findings reported in the original CASSEAL study [5], no significant differences were observed between senior and junior operators in visualization of any individual view or in overall completion of the 3 × 3 framework (Table 3). Visualization rates remained consistently high for both operators, exceeding 85% in most planes and approaching 100% for the conventional cardiac views. These findings suggest that the standardized sequential organization of the CASSEAL framework facilitates reproducible assessment of the EFCS between trained operators with different levels of overall fetal ultrasound experience.

Although Cohen’s kappa coefficients showed substantial variability across individual views, observed agreement remained consistently high throughout the examination (Table 3). This apparent discrepancy is well recognized in methodological literature and reflects the limitations of kappa statistics when category distributions are highly unbalanced, particularly when one outcome is overwhelmingly predominant [17,18]. Such situations may result in low or even negative kappa values despite high levels of observed agreement. Consequently, kappa coefficients should be interpreted in conjunction with percentage agreement, which remained high across all CASSEAL views in the present study. This interpretation was further supported by McNemar’s test, which showed no significant differences between operators for any individual CASSEAL view, indicating the absence of systematic operator-related bias.

Analysis according to EFCS territory revealed differences in diagnostic findings across the three anatomo-functional regions (Table 4). Four of the five true-positive examinations corresponded to ARSA within the supracardiac territory and were anatomically concordant with the final diagnosis. The remaining true-positive examination was anatomically discordant: an abnormality involving the cardiac outflow tract was suspected during first-trimester assessment, whereas follow-up identified a PRUV within the infracardiac territory. All four false-negative examinations corresponded to small ventricular septal defects. This distribution illustrates the different diagnostic challenges encountered across the three anatomo-functional territories of the EFCS. Vascular and mediastinal structures characterized by stable anatomical relationships may be particularly amenable to systematic evaluation using the CASSEAL framework, whereas subtle intracardiac defects remain inherently more difficult to identify during early gestation because of their small size and the limitations of first-trimester spatial resolution [1,16].

All four cases of ARSA were identified during first-trimester assessment. ARSA is a vascular variant and recognized sonographic marker rather than, in isolation, a major structural cardiac defect, and its clinical significance depends on the presence of associated chromosomal or structural abnormalities [11,12]. Its detection in the present study therefore primarily illustrates the ability of the CASSEAL framework to systematically visualize the supra-aortic vessels rather than providing evidence of sensitivity for major congenital heart disease. Conversely, all four false-negative examinations corresponded to small ventricular septal defects, which may have limited clinical significance but also illustrate the recognized limitations of first-trimester ultrasound for detecting small intracardiac lesions [1,16]. The anatomically discordant case in which an abnormal first-trimester CASSEAL examination was subsequently associated with a PRUV, together with the false-positive suspicion of PRUV in another fetus, further illustrates the diagnostic challenges of systematic assessment of the infracardiac territory. These findings likely reflect the small caliber of the portal venous structures during early gestation and the difficulty of distinguishing normal portal venous anatomy from subtle variants such as persistent right umbilical vein at 11–13 weeks of gestation. This territory has traditionally received less attention in conventional cardiac screening protocols despite its recognized clinical relevance [14].

From a clinical perspective, diagnostic performance of the CASSEAL algorithm in the first trimester was characterized by high specificity and negative predictive value (Table 5). However, predictive values are prevalence-dependent, and the low prevalence of confirmed EFCS abnormalities in this predominantly low-risk cohort (9/288, 3.1%) contributed to the high NPV observed. Conversely, the limited number of abnormal cases resulted in substantial uncertainty around sensitivity and PPV, as reflected by their wide confidence intervals. Likelihood ratios, which are less directly dependent on disease prevalence, provided complementary information; however, particularly for LR+, the wide confidence interval reflects the small number of abnormal and false-positive cases. These estimates should therefore be interpreted cautiously, also considering the absence of systematic postnatal echocardiographic verification in screen-negative infants. Consequently, the present study was not powered to provide a definitive assessment of diagnostic accuracy. Rather, its principal findings support the feasibility and reproducibility of structured first-trimester EFCS assessment, while diagnostic performance requires confirmation in larger cohorts with a broader spectrum of abnormalities and more uniform reference-standard verification.

Published approaches to first-trimester fetal cardiac screening remain heterogeneous, ranging from assessment of the four-chamber view alone to more comprehensive protocols incorporating outflow-tract views and color Doppler. A systematic review by Karim et al. demonstrated a progressive improvement in detection rates as the anatomical assessment became more comprehensive, particularly with the addition of outflow-tract views and color-flow Doppler [19]. However, these approaches remain primarily centered on cardiac anatomy. In contrast, the CASSEAL 3 × 3 framework was specifically designed to extend structured assessment beyond the heart itself by integrating infracardiac, cardiac, and supracardiac territories into nine standardized views. Thus, direct comparison of diagnostic performance between CASSEAL and previously published first-trimester cardiac screening protocols is limited by differences in anatomical scope and target abnormalities.

Comparison with the original CASSEAL study published in 2015 [5] provides valuable insight into the evolution of the framework over the last decade (Table 6). The original study demonstrated the feasibility and reproducibility of a structured nine-view sequential examination of the fetal cardiovascular system during the second trimester. Subsequent investigations expanded the anatomical scope of this approach through evaluation of additional cardiovascular territories, including the fetal thymus, supra-aortic vessels, aortic arch anatomy, venous circulation, and their integration within the concept of the Extended Fetal Cardiovascular System [6,9,10,11,12,13,14]. More recently, anatomical feasibility of applying the CASSEAL framework during the first trimester was demonstrated [15]. The present study represents the next step in this progression by providing the first clinical validation of the CASSEAL 3 × 3 framework during first-trimester assessment of the EFCS.

Despite being performed approximately seven weeks earlier in gestation than the original study, the CASSEAL framework maintained high overall feasibility and visualization rates across all nine views (Table 6). This achievement was obtained at the cost of longer examination times and more frequent use of complementary techniques, particularly color Doppler and transvaginal ultrasound. However, these adaptations enabled systematic assessment of the three EFCS territories during a developmental stage in which cardiovascular structures measure only a few millimeters. Taken together, these findings suggest that the CASSEAL framework can be successfully translated from second-trimester evaluation to early pregnancy while preserving its fundamental principles of structured, sequential, and comprehensive cardiovascular assessment. Despite these encouraging results, the mean examination time of approximately 12 min and the frequent need for complementary techniques suggest that implementation of the full CASSEAL framework may be more appropriate within specialized fetal medicine settings or as an adjunct to first-trimester screening rather than as a universal component of routine examinations. Future studies should evaluate strategies to optimize workflow and determine the feasibility of broader integration into routine screening programs.

This study has several limitations that should be considered when interpreting the results. The single-center design and the relatively small number of confirmed EFCS abnormalities limit the precision of diagnostic performance estimates, particularly sensitivity, resulting in relatively wide confidence intervals. The predefined exclusion of fetuses with nuchal translucency above the 95th percentile, major malformations, hydrops, or marked anatomical distortion also resulted in underrepresentation of pregnancies at highest risk of congenital cardiovascular abnormalities. Consequently, the spectrum of confirmed abnormalities was narrow and predominantly comprised ARSA and small VSDs. The diagnostic performance estimates obtained in this cohort should therefore not be extrapolated to high-risk populations, in whom both the prevalence and spectrum of cardiovascular abnormalities are expected to differ. Further validation in larger cohorts, including high-risk pregnancies and a broader range of major cardiovascular abnormalities, is required.

A further limitation concerns recruitment. Approximately one-third of women attending the first-trimester clinic could not be offered participation because their scheduled appointment occurred during clinical sessions not covered by the investigator. Recruitment therefore depended on investigator availability rather than on maternal, fetal, or ultrasound characteristics. Nevertheless, because systematic comparative data were not collected for non-participants, potential selection bias cannot be completely excluded, and the analyzed cohort may not fully represent the entire population attending the first-trimester clinic.

First-trimester ultrasound has inherent limitations for the detection of small intracardiac lesions, particularly ventricular septal defects, which remain highly dependent on spatial resolution and ongoing cardiac development. This limitation likely contributed to the false-negative findings observed within the cardiac territory and should be considered when interpreting the diagnostic performance of the CASSEAL framework.

The low prevalence of non-visualized views affected interpretation of Cohen’s kappa coefficients in several planes. This is a well-recognized methodological phenomenon that occurs when category distributions are highly unbalanced, potentially resulting in low kappa values despite high levels of observed agreement [17,18].

Furthermore, although the examination protocol proved feasible, successful completion of the full CASSEAL framework required longer examination times and selective use of complementary techniques, including color Doppler and transvaginal ultrasound. These requirements reflect the broader anatomical scope of EFCS assessment and may influence its implementation beyond specialized fetal medicine settings. The examinations in this study were performed in a tertiary fetal medicine center by operators with substantial experience in first-trimester fetal cardiovascular imaging. Therefore, the visualization rates, examination times, and diagnostic performance observed in this cohort may not be directly reproducible in lower-volume settings or among operators with less experience in early fetal cardiac assessment. Although the structured nature of the CASSEAL 3 × 3 framework is intended to facilitate standardized acquisition, its reproducibility across different levels of operator expertise was not specifically evaluated. A formal learning-curve analysis was not performed; while the senior operator had previous experience with the CASSEAL 3 × 3 framework, the junior operator did not. Therefore, the potential influence of operator experience and learning on visualization rates and examination time cannot be excluded. Future multicenter implementation studies should evaluate performance across different levels of operator expertise and determine the training requirements and learning curve necessary to achieve adequate visualization of the nine standardized views.

An additional limitation concerns the composite reference standard. Although all ongoing pregnancies underwent detailed second-trimester anatomical assessment and all newborns underwent routine neonatal clinical examination, postnatal echocardiography was not systematically performed in screen-negative infants but was reserved for cases with prenatal suspicion, abnormal neonatal findings, or other clinical indications. Consequently, minor or clinically silent abnormalities, including small VSDs, ARSA, or subtle venous variants, may have remained undetected among screen-negative cases. This differential or partial verification may have resulted in overestimation of specificity and, particularly, negative predictive value. Accordingly, the high NPV observed in this study should be interpreted in the context of the follow-up strategy and should not be considered equivalent to exclusion of EFCS abnormalities by systematic postnatal echocardiographic verification.

Taken together, these findings support the feasibility and reproducibility of the CASSEAL 3 × 3 framework for structured assessment of the Extended Fetal Cardiovascular System during the first trimester. The framework enables systematic evaluation of infracardiac, cardiac, and supracardiac territories within a unified examination, although its implementation requires additional examination time and frequent use of complementary imaging techniques. The diagnostic findings observed in this study should be considered preliminary given the limited number and spectrum of confirmed abnormalities and the characteristics of the reference standard. Further multicenter validation in larger and more diverse populations, including pregnancies at increased risk of fetal cardiovascular abnormalities, is required to determine its diagnostic performance and define its role within first-trimester screening.

## Figures and Tables

**Table 1:** Maternal characteristics, first-trimester ultrasound parameters, visualization quality of the axial views, and perinatal outcomes of the study cohort.

Variable			Mean ± SD; (%)	
Maternal				
Maternal age (years)			33.70 ± 5.08	
BMI (kg/m^2^)			24.97 ± 4.78	
Uterine anomaly (Yes)			20 (6.94)	
GA at US (weeks)			13.06 ± 0.69	
CRL (mm)			67.75 ± 8.45	
Time for the exploration (min)			12.49 ± 3.31	
Use of both (probes)			156 (54.55)	
Global algorithm feasibility			249 (86.46)	
CASSEAL Axial view	Visualization	Vaginal probe	Doppler	Normal view
Axial view I: “Portal sinus”	277 (96.18)	118 (40.97)	35 (12.15)	277 (96.18)
Axial view II: “Ductus venosus”	284 (98.61)	100 (34.72)	259 (89.93)	283 (98.26)
Axial view III: “Hepatic Veins”	263 (91.32)	115 (39.93)	253 (87.85)	261 (90.63)
Axial view IV: “Four-chamber view”	285 (98.96)	119 (41.32)	17 (5.90)	285 (98.96)
Axial view V: “Left ventricular outflow tract”	285 (98.96)	129 (44.79)	45 (15.63)	284 (98.61)
Axial view VI: “Right ventricular outflow tract”	281 (97.57)	128 (44.44)	68 (23.61)	281 (97.57)
Axial view VII: “Three-vessel and trachea view”	284 (98.61)	129 (44.79)	206 (71.53)	284 (98.61)
Axial view VIII: “Thy-box”	271 (94.10)	119 (41.32)	288 (100)	271 (94.10)
Axial view IX: “Subclavian arteries”	281 (97.57)	132 (45.83)	274 (95.14)	276 (95.83)
Perinatal				
Stillbirth			2 (0.69)	
Cesarean section			72 (25.35)	
Gestational age at delivery (weeks, calculated)			38.72 ± 4.91	
Newborn weight (g)			3194 ± 465	
Apgar score at 1 min			8.6 ± 1.4	
Apgar score at 5 min			9.7 ± 1.1	
Umbilical cord arterial pH			7.25 ± 0.08	

Data are presented as mean ± standard deviation (SD) or *n* (%), as appropriate. Visualization indicates successful acquisition of the corresponding CASSEAL view. Vaginal probe refers to cases in which transvaginal ultrasound was required to obtain the view. Doppler indicates use of color Doppler imaging during assessment of the corresponding plane. Normal view refers to cases fulfilling the predefined normality criteria for each CASSEAL view. Global algorithm feasibility was defined as successful visualization of all nine CASSEAL axial views. Abbreviations: BMI, body mass index; GA, gestational age; US, ultrasound examination; CRL, crown–rump length; SD, standard deviation.

**Table 2:** Factors associated with complete visualization of the nine CASSEAL axial views: univariable and multivariable analyses.

Variable	OR (CI95) Univariable	*p* Value	OR (CI95) Multivariable	*p* Value
Maternal age (year)	1.06 (0.99–1.14)	0.95	1.06 (0.99–1.15)	0.11
Gestational age at ultrasound (weeks)	2.44 (1.45–4.17)	<0.001	2.44 (1.45–4.35)	<0.001
BMI (kg/m^2^)	0.91 (0.85–0.96)	<0.01	0.91 (0.85–0.97)	<0.01
Uterine anomalies	1.45 (0.40–9.09)	0.61	0.85 (0.21–5.88)	0.85

Odds ratios (ORs) and 95% confidence intervals (95% CI) were obtained from univariable and multivariable logistic regression analyses. Complete visualization of the CASSEAL 3 × 3 framework (all nine axial views successfully visualized) was used as the dependent variable. OR > 1 indicates an increased likelihood of complete visualization, whereas OR < 1 indicates a reduced likelihood of complete visualization. Abbreviations: OR, odds ratio; CI, confidence interval; BMI, body mass index.

**Table 3:** Comparison of first-trimester CASSEAL axial view visualization between senior and junior operators: visualization rates, agreement and Cohen’s kappa.

View	Senior (%)	Junior (%)	*p*-Value	Percentage Agreement	Kappa	*p* McNemar
Global algorithm feasibility	74.0	62.0	0.146	76.0	0.458	0.146
Axial view I: “Portal sinus”	92.0	86.0	0.508	82.0	0.089	0.508
Axial view II: “Ductus venosus”	100.0	98.0	1.000	98.0	0.000	1.000
Axial view III: “Hepatic Veins”	88.0	84.0	0.754	80.0	0.172	0.754
Axial view IV: “Four-chamber view”	100.0	100.0	N/A	100.0	N/A	—
Axial view V: “Left ventricular outflow tract”	100.0	98.0	1.000	98.0	0.000	1.000
Axial view VI: “Right ventricular outflow tract”	94.0	98.0	0.500	96.0	0.485	0.500
Axial view VII: “Three-vessel and trachea view”	100.0	96.0	0.500	96.0	0.000	0.500
Axial view VIII: “Thy-box”	90.0	90.0	1.000	88.0	0.333	1.000
Axial view IX: “Subclavian arteries”	98.0	88.0	0.125	86.0	−0.036	0.125

Data are expressed as the percentage of examinations in which each CASSEAL view was successfully visualized by the senior and junior operators. Percentage agreement represents the proportion of cases with concordant classifications between observers. Cohen’s kappa coefficient was used to assess interobserver agreement beyond chance. Low or null kappa values in some views should be interpreted in the context of highly unbalanced distributions with visualization rates approaching 100%. McNemar’s test was used to evaluate whether discordant classifications were systematically biased toward either operator. Abbreviations: N/A: Kappa could not be calculated due to zero variance in a 100% agreement matrix.

**Table 4:** Patient-level classification and follow-up of suspected or confirmed EFCS abnormalities.

Case	First-Trimester CASSEAL Finding	Final Diagnosis	Classification
1	ARSA	ARSA	True positive
2	ARSA	ARSA	True positive
3	ARSA	ARSA	True positive
4	ARSA	ARSA	True positive
5	Suspected outflow tract anomaly	PRUV	True positive *
6	Normal	VSD	False negative
7	Normal	VSD	False negative
8	Normal	VSD	False negative
9	Normal	VSD	False negative
10	Suspected PRUV	Normal	False positive

* Case 5 was classified as a true positive at the patient level because both the first-trimester CASSEAL examination and the reference standard indicated the presence of an EFCS abnormality. However, the specific anatomical finding suspected at first-trimester examination was not confirmed, and follow-up identified a PRUV instead. Abbreviations: ARSA, aberrant right subclavian artery; VSD, ventricular septal defect; PRUV, persistent right umbilical vein; EFCS, Extended Fetal Cardiovascular System.

**Table 5:** Patient-level diagnostic performance of the first-trimester CASSEAL algorithm using the composite prenatal and postnatal reference standard.

Variable	Overall
True positives (TP)	5
False positives (FP)	1
False negatives (FN)	4
True negatives (TN)	278
Sensitivity (95% CI)	55.56% (21.20–86.30)
Specificity (95% CI)	99.64% (98.02–99.99)
PPV (95% CI)	83.33% (35.88–99.58)
NPV (95% CI)	98.58% (96.41–99.61)
Positive likelihood ratio (LR+)	155.0 (20.12–1194.30)
Negative likelihood ratio (LR−)	0.45 (0.21–0.93)

Diagnostic performance measures were calculated at the patient level using the composite prenatal and postnatal reference standard. Sensitivity, specificity, positive predictive value (PPV), and negative predictive value (NPV) are presented with their corresponding 95% confidence intervals (95% CI). Diagnostic classification was based on the presence or absence of any EFCS abnormality, irrespective of anatomical concordance between the first-trimester suspected finding and the final diagnosis. Given the limited number of confirmed EFCS abnormalities, diagnostic performance estimates, particularly sensitivity, should be interpreted with caution. Positive and negative likelihood ratios (LR+ and LR−) are also presented with 95% confidence intervals. Given the limited number of confirmed EFCS abnormalities, particularly the small number of false-positive cases, likelihood-ratio estimates should also be interpreted cautiously. Abbreviations: PPV, positive predictive value; NPV, negative predictive value; CI, confidence interval; EFCS, Extended Fetal Cardiovascular System.

**Table 6:** Comparison between the original unicenter JUM 2015 study [15] and the current study.

Variable	Single-Center JUM 2015 [15]	Current Study
GA at US	20.0 ± 0.6 weeks	13.1 ± 0.7 weeks
Gestational trimester	Second trimester	First trimester
Operators	2 (senior vs. junior)	2 (senior vs. junior)
Fetuses scanned	184	288
Study type	Single center	Single center
Axial view I: “Portal sinus”	100%	96.18%
Axial view II: “Ductus venosus”	99.5%	98.61%
Axial view III: “Hepatic Veins”	98.9%	91.32%
Axial view IV: “Four-chamber view”	99.5%	98.96%
Axial view V: “Left ventricular outflow tract”	99.5%	98.96%
Axial view VI: “Right ventricular outflow tract”	99.5%	97.57%
Axial view VII: “3-vessel and trachea view”	99.5%	98.61%
Axial view VIII: “Thy-box”	88.7%	94.10%
Axial view IX: “Subclavian arteries”	98.4%	97.57%
Global algorithm feasibility	81.5%	86.5%
Mean scan time	5.6 ± 4.2 min	12.5 ± 3.3 min
Scan time ≤ 10 min	92.5%	16.31%
Doppler use (global)	Frequent as complementary technique	Variable and view-dependent
Doppler use per view	Reported in aggregate	Quantified per view
Use of vaginal probe	0	Between 35–48% depending on view
Inter-observer agreement (kappa)	High (except view VIII)	Variable; moderate for global
Factors associated with feasibility	Fetal position (occiput anterior)	Maternal and technical factors
Fetal anomalies	Excluded from analysis	Included in analysis
Perinatal outcomes	Reported	Reported
Main objective	Feasibility and reproducibility	Feasibility, quality, agreement, and applicability

Comparison between the original CASSEAL study and the present first-trimester validation study. The table summarizes differences in study design, gestational age at examination, anatomical targets, feasibility outcomes, and clinical objectives. Direct comparisons should be interpreted cautiously because of differences in study populations, gestational age ranges, and methodological objectives. Abbreviations: EFCS, Extended Fetal Cardiovascular System; GA, gestational age.

## Data Availability

The data presented in this study are available from the corresponding author upon reasonable request. The data are not publicly available due to privacy and ethical restrictions.

## Supplementary Materials

The following supporting information can be downloaded at: https://www.mdpi.com/article/10.3390/jcdd13090422/s1, Table S1: Normality criteria for the nine standardized views of the CASSEAL 3 × 3 framework during first-trimester assessment of the Extended Fetal Cardiovascular System (EFCS).

## Abbreviations

The following abbreviations are used in this manuscript:

ARSA	Aberrant Right Subclavian Artery
BMI	Body Mass Index
CASSEAL	Cardiovascular System Sonographic Evaluation Algorithm
CHD	Congenital Heart Disease
CI	Confidence Interval
CRL	Crown–Rump Length
EFCS	Extended Fetal Cardiovascular System
GA	Gestational Age
NPV	Negative Predictive Value
OR	Odds Ratio
PPV	Positive Predictive Value
PRUV	Persistent Right Umbilical Vein
SD	Standard Deviation
US	Ultrasound
VSD	Ventricular Septal Defect
