# Supplementary material for: First-Trimester Assessment of the Extended Fetal Cardiovascular System Using the CASSEAL 3 × 3 Framework: Feasibility, Reproducibility and Diagnostic Performance
Source: J Cardiovasc Dev Dis. 2026 Sep 1;13(9):422. doi: 10.3390/jcdd13090422 (PMC13607861; doi:10.3390/jcdd13090422)
Supplement: Supplementary file 1 [file jcdd-13-00422-s001.zip › jcdd-4410739-supplementary.pdf]

Supplementary Table S1. Normality criteria for the nine standardized views of the CASSEAL 3×3 framework during first-trimester assessment of the Extended Fetal Cardiovascular System (EFCS).

**Territory**	**View**	**Anatomical structure**	**Criteria for normality**
Infracardiac	I	Portal sinus	Normal abdominal situs; visualization of the portal sinus with normal course and relationship to the umbilical vein.
Infracardiac	II	Ductus venosus	Identification of the ductus venosus connecting the umbilical vein to the inferior vena cava with normal antegrade Doppler flow.
Infracardiac	III	Hepatic veins	Visualization of hepatic venous drainage into the inferior vena cava with normal anatomical relationships.
Cardiac	IV	Four-chamber view	Presence of four cardiac chambers of similar size, normal cardiac axis, intact crux, and normal situs.
Cardiac	V	Left ventricular outflow tract (LVOT)	Continuity between the left ventricle and ascending aorta with normal alignment and antegrade flow.
Cardiac	VI	Right ventricular outflow tract (RVOT)	Continuity between the right ventricle and pulmonary artery with normal crossing relationship and antegrade flow.
Supracardiac	VII	Three-vessel and trachea view (3VT)	Visualization of the pulmonary artery, aorta, and superior vena cava with normal arrangement and relative vessel size.
Supracardiac	VIII	Thymic box (thy-box)	Presence of thymic tissue within the thy-box boundaries with normal appearance and symmetry.
Supracardiac	IX	Subclavian vessels	Visualization of right and left subclavian vessels with normal origin, course, and symmetry.
